# Use of Methylene Blue in the Treatment of Refractory Vasodilatory Shock After Cardiac Assist Device Implantation: Report of Four Consecutive Cases

**DOI:** 10.4021/jocmr804w

**Published:** 2012-05-15

**Authors:** Sebastian Michel, Florian Weis, R Sodian, Andres Beiras-Fernandez, Amir K Bigdeli, Ingo Kaczmarek, Dirk Bruegger

**Affiliations:** a Department of Cardiac Surgery, Ludwig-Maximilians-University, Munich, Germany; b Department of Anesthesiology, Ludwig-Maximilians-University, Munich, Germany

**Keywords:** Methylene blue, Vasodilatory shock, Assist device

## Abstract

Vasodilatory shock frequently occurs after cardiac surgery, particularly after cardiac assist device implantation. This complication is often associated with high mortality, especially if refractory to conventional vasoconstrictor treatment. Methylene blue, a guanylate cyclase inhibitor, has been successfully used in the management of vasodilatory shock associated with cardiopulmonary bypass. We present four successive cases after implantation of cardiac assist devices suffering from norepinephrine and vasopressin refractory severe vasodilatory shock. In all patients, administration of a single dose of methylene blue (2 mg/kg body weight) resulted in an immediate and persistent decrease in vasoconstrictor dosages and serum lactate concentrations. Despite of this benefit, all patients deceased during hospital stay, however, this was not related to the methylene blue treatment. Methylene blue seems to be a promising therapeutical option in patients with otherwise resistant vasodilatory shock after cardiac assist device implantation. However, controlled clinical trials are necessary to substantiate safety and efficacy.

## Introduction

Vasodilatory shock is a frequent complication in the postoperative period in patients undergoing cardiac surgery which increases morbidity and mortality. It is characterized by peripheral vasodilatation, severe hypotension and by a poor response to therapy with vasoconstrictors. One important pathophysiological facet of this disease is an increased synthesis of nitric oxide [1, 2]. The use of the guanylate cyclase inhibitor methylene blue has been shown to be a successful therapeutical option in vasoplegic cardiac surgical patients undergoing coronary artery bypass surgery or valve replacement [3]. After implantation of cardiac assist devices, the incidence of vasodilatory shock has been described to be excessively high [4]. We report four successive patients after cardiac assist device implantation with catecholamine refractory vasoplegia, who were successfully treated with a single dose of methylene blue without any immediate serious side effects.

## Case Report

All patients received a single dose infusion of methylene blue (2 mg/kg body weight intravenously) over 30 minutes.

### Case 1

**Figure 1:**
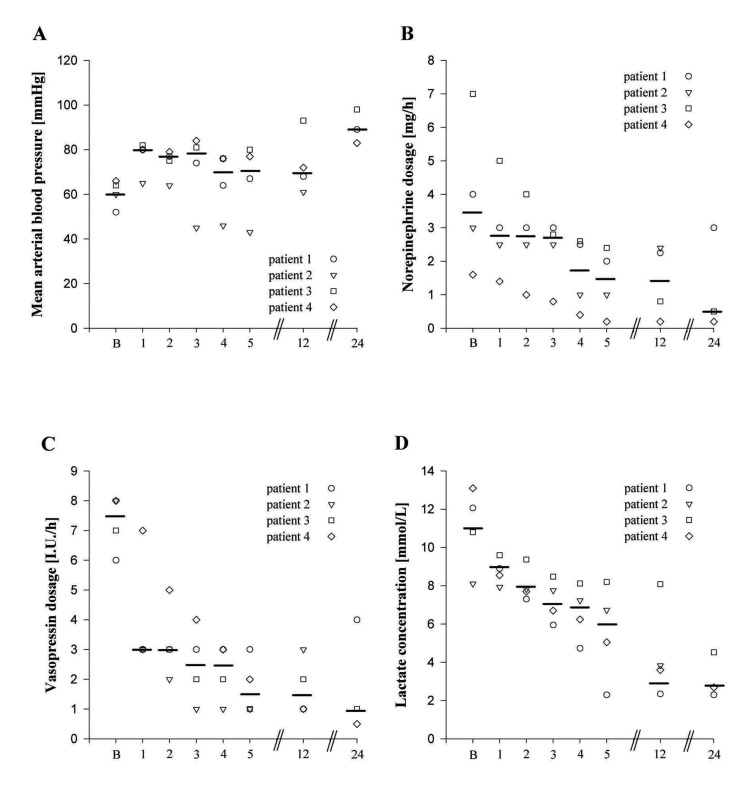
Individual and median values of mean arterial blood pressure (panel A), norepinephrine dosage (panel B), vasopressin dosage (panel C), and serum lactate concentration (panel D) before (B) and 1, 2, 3, 4, 5, 12, and 24 hours after a single dose administration of methylene blue (2 mg/kg body weight).

A 49-year-old male patient (188 cm, 98 kg) suffered from decompensated dilative cardiomyopathy. As he did not respond to conventional treatment (inotropes and insertion of an intraaortic balloon pump) a Jarvik 2000 left ventricular assist device (Jarvik Heart Inc., NYC, NY, USA) was implanted. Postoperatively, the patient developed right heart failure, necessitating the implantation of an additional temporary right ventricular assist device (Levitronix, Levitronix GmbH, Zurich, Switzerland). Severe postoperative bleeding required rethoracotomy and mass transfusion. Despite no further bleeding and faultless function of both cardiac assist devices, the patient developed a severe vasoplegic shock, requiring very high dosages of norepinephrine (4.0 mg/h) and vasopressin (6.0 I.U./h). After administration of a single dose methylene blue both norepinephrine and vasopressin dosages rapidly decreased and serum lactate concentrations normalized (Fig. 1). On the fourth postoperative day the patient developed pneumonia caused by pseudomonas aeruginosa with severe impairment of pulmonary function. An oxygenator was connected to the right ventricular assist device to enable sufficient oxygenation. Despite inhalative application of milrinone and iloprost, right ventricular function did not recover sufficiently. Although the patient was treated with antibiotics (piperacillin/tazobactam, ciprofloxacin, vancomycin) markers of infection did not decrease and the patient died ten days after methylene blue administration from multi-organ failure.

### Case 2

A 46-year-old male patient (185 cm, 80 kg) with a history of myocardial infarction and limited cardiac function (LVEF 20%) underwent resection of a massive left ventricular anterior wall aneurysm combined with mitral valve annuloplasty. Despite adequate filling pressures, use of multiple inotropic agents including levosimendan and insertion of an intraaortic balloon pump, cardiogenic shock occured postoperatively. He was transferred to the operating room for venoarterial extracorporal membrane oxygenation (ECMO, Medtronic, Biomedicus, MI, USA) rescue therapy. Since weaning from ECMO remained unsuccessful for four days, a permanent left ventricular assist device (Jarvik 2000) was inserted. Postoperatively, the patient suffered from right heart failure resistant to inhalation of iloprost and milrinone, so that venoarterial ECMO had to be re-implanted. Despite good function of both cardiac assist devices the patient suffered from severe vasodilatory shock resistant to high dosages of norepinephrine (3.0 mg/h) and vasopressin (8.0 I.U./h). Administration of methylene blue resulted in a reduction of norepinephrine and vasopressin to acceptable dosages within four hours (Fig. 1). However, no therapeutic option was suitable for this patient: switching to a synchronized biventricular assist device was technically not possible any more. The patient died 18 hours after methylene blue administration.

### Case 3

In a 32-year-old male patient (187 cm, 114 kg) with decompensated dilative cardiomyopathy a biventricular assist device (Berlin Heart Excor, Berlin Heart GmbH, Berlin, Germany) was implanted, as a bridge to transplant. Postoperatively, severe septic shock caused by pneumonia occurred and was treated empirically with meropenem, moxifloxacin, and linezolid. Despite adequate filling pressures, 7.0 mg/h of norepinephrine and 7.0 I.U./h of vasopressin were necessary to stabilize circulatory conditions. Methylene blue was applicated and vasoconstrictor dosages could be reduced within hours (Fig. 1). On the fourth postoperative day the patient suddenly developed pulmonary edema. Despite therapeutical heparinization and aspirin therapy echocardiography revealed a thrombus, most probably derived from the left atrial appendage, nearly completely occluding the left ventricular inflow cannula. In an immediate re-operation the left ventricular inflow cannula was replaced from the left ventricular apex to the left atrium. During surgery, the left atrium ruptured. Surgical repair was unfeasible, so that the patient died intraoperatively from severe hemorrhage.

### Case 4

A 70-year-old male patient (155 cm, 64 kg) with pre-existing coronary heart disease underwent coronary angiography with instable angina pectoris. A significant stenosis of the right coronary artery was detected. The attempt to insert the wire into the right coronary artery resulted in a complete dissection of the vessel. Emergency coronary artery bypass operation with a saphenous vein graft was performed immediately, but revascularisation was technically impossible. Accordingly, the patient developed right heart failure and received venoarterial ECMO. Postoperatively, norepinephrine (1.6 mg/h) and vasopressin (8.0 I.U. /h) resistant vasodilatory shock occurred. After a single dose of methylene blue vasoconstrictor dosages decreased within hours (Fig. 1). Despite maximum pharmacological support including inhalation of iloprost and milrinone right ventricular performance could not be improved during the following seven days. As the patient was considered to be too old for implantation of a definitive right ventricular assist device, therapeutic measures were terminated. The patient died 8 days after methylene blue administration.

## Discussion

The present case series shows that intravenous single dose administration of methylene blue in patients with vasodilatory shock after cardiac assist device implantation resulted in improved hemodynamics. An immediate and persistent decrease of vasopressor (norepinephrine and vasopressin) dosages was observed in all patients. Moreover, the lower lactate levels after infusion suggest that methylene blue increased the vascular tone without compromising global tissue perfusion (Fig.1).

One underlying mechanism of vasodilatory shock involves increased nitric oxide production. The release of nitric oxide leads to activation of the enzyme guanylate cyclase, which increases the synthesis of cGMP, a mediator of vascular smooth muscle relaxation [1]. Methylene blue inhibits guanylate cyclase, thus, mitigating the vasorelaxant effect of nitric oxide in the smooth muscle cells of the vessels [2, 5].

At the time point of methylene blue administration all patients were severely ill with multiple organ failure and high Apache II scores ranging between 32 and 40 with a predicted mortality between 76% and 91% [6]. Despite the beneficial effects on hemodynamics and serum lactate concentrations all patients died during hospital stay. However, this was not related to methylene blue administration. At a dosage of 2 mg/kg body weight no serious side effects were observed. The only adverse effects were discolouration of the urine (greenish-blue) in all four patients and shivering in one patient (patient 2).

We conclude that methylene blue, through its effect on the nitric oxide pathway, is a promising option in the treatment of patients with vasodilatory shock after cardiac assist device implantation. However, a controlled clinical trial is needed to evaluate the benefit and best time point of application of methylene blue in severely ill patients before routine use can be recommended.
